# Ovary Cells Apoptosis in Opium-Addicted Diabetic and Non-Diabetic Rats

**DOI:** 10.5812/ijhrba.8409

**Published:** 2013-06-26

**Authors:** Gholamreza Asadikaram, Majid Asiabanha, Majid Sirati Sabet

**Affiliations:** 1 Physiology Research Center, Kerman University of Medical Sciences. Kerman, IR Iran; 2 Department of Biochemistry, Kerman University of Medical Sciences, Kerman, IR Iran; 3 Department of Biochemistry and Genetic, Faculty of Medicine, Qazvin University of Medical Sciences, Qazvin, IR Iran

**Keywords:** Addiction, Diabetes, Opium, Apoptosis, Ovary

## Abstract

**Background:**

Apoptosis is a physiological mechanism of cell death and it can be triggered by a variety of internal and external stimuli. It has been indicated that some opium derivatives develop cell apoptosis.

**Objectives:**

The aim of this investigation was to evaluate the effect of opium addiction on ovary cell apoptosis in diabetic and non-diabetic Wistar rats.

**Materials and Methods:**

This experimental study was done on control, control-addicted, diabetic and diabetic-addicted rats. DNA fragmentation as a biomarker of apoptosis was determined by the TUNEL assay.

**Results:**

The blood glucose concentration in diabetic-addicted and diabetic rats was increased when compared to control (P < 0.001). There was no significant difference between weights of control, control-addicted (non-diabetic) and diabetic-addicted groups during this study. The results of this study indicated that apoptosis in addicted and diabetic-addicted ovary cells was significantly higher than in diabetic group, and also apoptosis in addicted group was significantly more than the control rats. In addition, we found that ovary cells apoptosis of diabetic rats were significantly less than in control group.

**Conclusions:**

Overall, these findings suggest that opium-addiction could play an important role in ovary cell apoptosis and could be very harmful for the reproductive system. Also, ovary cells of non-diabetic rats are more susceptible to opium-induced apoptosis than those of diabetic.

## 1. Background

Apoptosis or programmed cell death is known as both a normal process and a pathological feature in cell development. The apoptotic process can be triggered by a variety of internal and external stimuli (1). Opium contains 8 - 17% morphine, 1 - 10% noscapnie, 0.5 - 15% papaverine and 0.7 - 5% codeine and is used as a raw material in production of the mention alkaloids (2). Consumption of opium can effect some biochemical factors (3, 4), transforming growth factor β (TGF-β) (5), and causes brain and liver cells’ apoptosis (6). Considerable evidence is available suggesting that morphine (7-9), noscapine (10-13), codeine (14) and papaverine (15) have apoptotic activity *in vitro* and *in vivo*. Thus, the effect of opium on apoptosis and cell function could be different from pure drugs such as morphine, noscapine, codeine and papaverine because more than 20 alkaloids (16) and more than 70 constituents (17), have been detected in opium. The influence of opium components on apoptosis and changes in GnRH and LH secretion (18), led us to investigate the effects of opium on apoptosis in the ovary. Since some people believe opium has therapeutic effects on many disorders including diabetes mellitus (3, 4) and the fact that many female reproductive problems occur in diabetes (19, 20), we extended the study to diabetic animals.

## 2. Objectives

This study was designed to examine the impact of opium addiction on ovary cell apoptosis in diabetic and non-diabetic Wistar rats.

## 3. Materials and Methods

Opium was obtained from the anti-drug section of Kerman Police (Iran) and the origin of it was Helmand in Afghanistan. GC-mass spectrometry analysis of the opium used in the present study indicated that it consisted of more than 30% alkaloids (in which morphine 16%, codeine 5.5%, the baine 4.4% and papaverine 3.2% were the most abundant) and the rest consisted of non-alkaloidal organic and non-organic substances in which 13.5% was water (moisture). TUNEL (terminal deoxynucleotidyl transferase (TdT)-mediated dUTP-X nick end labeling) kit was obtained from Roche Diagnostic (Manheim, Germany). Proteinase K was purchased from Roche, Germany. Streptozocin (STZ) was procured from the Pfizer Company (AG, Zurich, Switzerland). DNA extraction kit was obtained from Cinnagen, Iran. Glucose oxidase kit was also acquired from the Pars Azemoon Company (Tehran, Iran). All other analytical grade laboratory chemicals and reagents were purchased from Merck or Sigma. In this experimental study, 28 female Wistar rats (250 - 300 g) were selected and divided into four groups. 7 female rats were used as controls and 14 were used for induced diabetics. Animals were housed in stainless steel cages in at temperature-controlled room (22°C) with a 12-h light/dark cycle and had free access to food and water. All animal experiments were carried out in accordance with the European Communities Council directive of 24 November 1986 (86/609/EEC) in a way to minimize the number of animals and their suffering. Diabetes was induced by a single dose of STZ (60 mg/kg of the body weight), dissolved in 0.1 M sodium citrate buffer (pH 4.4) that was intravenously injected into the tail vein. Control rats were injected with vehicle buffer only. Blood samples were obtained from orbit cavity under ether anesthesia using a thin heparinized tube at 72 h after STZ injection and plasma glucose levels were determined by the glucose oxidase method (2100 spectrophotometer; Unico, USA). Rats with glucose levels above 250 mg/dl were used as diabetics (21). 7 of the diabetic animals along with 7 of the control (non diabetic) animals were treated with a double dose (8 AM and 8 PM) of opium, which was dissolved in fresh saline for 8 consecutive days by peritoneal injection. The protocol of treatment was as follows: on the first to fifth day 30, 60, 90, 120, and 150 mg/kg was administered respectively. From the sixth to eighth day, 150 mg/kg dose was continued (this dose was the maximum tolerable dose for animals). The control groups received normal saline. Animals weight were determined at the ninth day of opium injection and after that, a single dose of 150 mg/kg opium was injected (5). The control groups received normal saline. After 3 hours, rats were decapitated under ether anesthesia. Finally the ovaries of 7 animals of each group were used for apoptosis evaluation. The withdrawal signs in opium-dependent rats were detected from the 5th day of opium injection. These signs which were occasionally studied for a short duration previous to the next injection were wet-dog shakes as the first sign, and then head shakes, irritability, hyperactivity, ptosis, and writhing (6). Immediately after the rats were killed, the ovaries were removed and embedded in 0.1 M phosphate-buffered saline (PBS) at pH 7.4 containing 3.7% paraformaldehyde for 30 minutes at room temperature. The tissues were incubated for 7.5 hours in 3.7% paraformaldehyde. Ovaries were then shifted to 75% ethanol and rinsed for 1 hour. Washing was then repeated with ethanol (75%, 95% and 100 %). The tissues were then transferred to xylenes and were rinsed twice for 1 hour. Tissues were fixed in molten paraffin wax (58 ºC) for 1 hour, and were then embedded again by fresh paraffin wax (6). The apoptotic nuclei DNA fragmentation was assessed by the TUNEL technique using the *in situ* cell death detection kit based on the manufacturer’s kit manual that has been described previously (6). The data are expressed as Mean ± SD. All analysis was performed using the SPSS software (version 18, SPSS Inc.). Analysis of data was done using the t-test and analysis of variance (ANOVA) to compare the mean values between the multiple groups. Statistical differences with P < 0.05 were considered as significant.

## 4. Results

**Figure 1.**
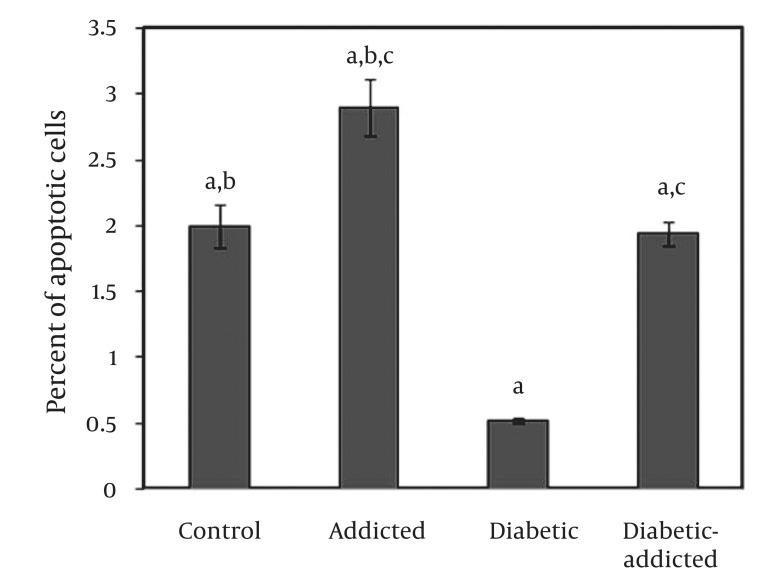
Comparison of Ovary Cells Apoptosis in Different Groups of the Study, (Values Are Mean ± SD) (a) Significant difference between diabetes and other groups (P < 0.01). (b) Significant difference between addicted and control group (P < 0.047). (c) Significant difference between addicted and diabetic-addicted (P < 0.01).

*Table 1* shows the changes in blood glucose concentration and body weights during this study. There was no significant difference between weights of control, addicted (non-diabetic) and diabetic-addicted rats. *Figure 1*, shows that there was a significant difference in apoptosis between diabetic (0.52% ± 0.04) and control (2.00% ± 0.33), (P < 0.01), addicted (2.90% ± 0.43) and control (2.00% ± 0.33), (P < 0.047), diabetic-addicted (1.94% ± 0.19) and addicted (2.90% ± 0. 43), (P < 0.01) and diabetic-addicted (1.94% ± 0.19) and diabetic (0.52% ± 0.04), (P < 0.01) groups. No significant difference in apoptosis was observed between the control (2.00% ± 0.33) and diabetic-addicted (1.94% ± 0.19) groups (*Figure 1*).

**Table 1.** Blood Glucose Levels and Weights of the Study Groups (Values Are Mean ± SD)

	Control	Control addicted	Diabetic	Diabetic addicted
**Initial body weight, g**	270 ± 21	265 ± 20	268 ± 22	271 ± 17
**Final body weight, g**	283 ± 19	273 ± 27	254 ± 25	256 ± 34
**Initial blood glucose, mg/dl**	107.4 ± 8.5	105.3 ± 5.8	106.0 ± 6.8	103.4 ± 7.9
**Final blood glucose, mg/dl**	105.8 ± 9.9	108.7 ± 8.5 ^a^	450.9 ± 57.5 ^a^	403.9 ± 46.8

^a^P < 0.001 versus control

## 5. Discussion

In the present study, we found that ovary cell apoptosis is induced by opium addiction in diabetic and non-diabetic rats. In addition, ovary cells of non-diabetic rats are more susceptible to induction of apoptosis than those of diabetic rats. Some people believe that opium has a positive therapeutic effect on some disorders (3), especially diabetes mellitus (4). Due to this basis, ordinary people recommend opium and that serves as a reason for its consumption. Based on a WHO report, more than 2.8% of the adult population of Iran is opium-addicted (22). Regarding the life threatening effects of morphine, they are generally expected to happen at high doses; in the present study we applied the maximum tolerance dose of opium (150 mg/kg). Although there are a large number of reports regarding the effects of opiate derivatives on cells apoptosis in the literature (7-9, 14, 19, 23), to our knowledge, this is the first study on the effects of opium on ovary cell apoptosis. In a previous study, we demonstrated that opium treatment can differentially induce brain and liver cell apoptosis in diabetic and non-diabetic male and female rats (6). A recent study on neurons and glial cells has proposed that chronic treatment with high doses of morphine stimulates apoptosis (8). In addition, it has been proven that chronic intra peritoneal treatment with morphine or heroin and heroin withdrawal produce up-regulation of the death receptor Fas and decreases the expression of the anti-apoptotic Bcl-2 onco-protein in the cerebral cortex of rats (7, 19, 23). Papaverine, another derivative of opium, is a vasodilator which is known to induce apoptosis in vascular endothelial and smooth muscle cells (15). Noscapine (one of the main components of opium) which serves as a tubulin-binding agent has also been demonstrated to activate micro-tubule dynamics to block mammalian cells mitosis through triggering of the c-Jun NH2-terminal kinase (JNK) pathway (11). It has been reported that C-Jun facilitates apoptosis of colorectal carcinoma P53 dependent pathway (10). It has been proven that apoptosis is triggered by intracellular signals or extrinsic death activators (1). The responsible agents of activation in each pathway have different results in male or female animals or tissues, suggesting that intrinsic differences are solely based on gender (6, 24). The results of the present study regarding opium-induced apoptosis in diabetic and non-diabetic rat ovaries are consistent with the above reports. On the other hand it has been shown that hyperglycemia, both in the short and long term, increases apoptosis in the granulose cells, up-regulates the extrinsic apoptotic pathway, and diminishes levels of a key gap junction protein (25). As shown in figure 1, ovary cells apoptosis in the control group is significantly higher than the diabetic rats. Our findings are not consistent with the above result (25), as ovary cells of non-diabetic rats are more susceptible to induction of apoptosis than those of diabetic rats. This paradox remains to be explained. Probably, pathogenesis of hyperglycemia needs more time than available in this study. However, diabetes-associated changes in ovarian follicular metabolism and structure, associated with pronounced hyperlipidemia, depressed ovarian steroid hormone biosynthesis, depressed sensitivity and responsiveness to hormones, suppressed maturation of follicles have been reported. These alterations lead to progressive accumulation of cytolipid inclusions, nuclear compartment seclusion, inhibition of cellular oxidative metabolism in ovarian follicles (19). Also it has been reported that in diabetes, apoptotic disruption of ovarian follicular granulosa cell layers is a coincident of nuclear lipid-infiltration and DNA fragmentation, events that are linked to the metabolic disturbances resulting from progressive hypercytolipidemia within the female reproductive tract (20). Hence, it could be concluded that changes in biological metabolic activity in diabetic-ovary cells may lead to alteration in the apoptosis process of ovary cells, and in these cases different results would be expected. In summary, our results showed that opium addiction could possibly impose damage on ovary cells by inducing apoptosis. In addition, ovary cells of non-diabetic rats are more susceptible to opium induced apoptosis than those of diabetic rats.
